# Considering the nutritional benefits and health implications of red meat in the era of meatless initiatives

**DOI:** 10.3389/fnut.2025.1525011

**Published:** 2025-01-27

**Authors:** Melissa Kavanaugh, Diana Rodgers, Nancy Rodriguez, Frédéric Leroy

**Affiliations:** ^1^Department of Epidemiology, College of Public Health, The University of Iowa, Iowa City, IA, United States; ^2^Global Food Justice Alliance, Boston, MA, United States; ^3^Department of Nutritional Sciences, University of Connecticut, Storrs, CT, United States; ^4^Research Group of Industrial Microbiology and Food Biotechnology, Faculty of Sciences and Bioengineering Sciences, Vrije Universiteit Brussel, Brussels, Belgium

**Keywords:** red meat, nutrient density, undernutrition, obesity, plant-source

## Abstract

Driven by perceived health and environmental benefits, initiatives to remove red meat from recommended eating patterns are increasingly being implemented in United States institutions, including schools and hospitals. While these efforts aim to address important issues, they may inadvertently lead to unintended consequences, particularly regarding essential nutrient intake for certain populations. This perspective considers the nutritional value of red meat, examines its potential health benefits, and highlights nutritional risks when intake is reduced or eliminated. Red meat is a nutrient-dense food that provides highly bioavailable protein and several essential micronutrients often lacking in the diet, including iron, zinc, and vitamin B_12_. These nutrients can be limited or absent in many plant-source foods as well as in some animal-source foods. Red meat’s micronutrient profile is especially valuable for young children and women of reproductive age, including pregnant women. In addition, the protein density of red meat is beneficial for individuals managing obesity and older adults at risk of sarcopenia. Many epidemiological studies suggest potential associations between excessive red meat consumption and increased risk of certain chronic diseases, but this evidence does not conclusively implicate red meat in the development or progression of chronic disease. The nutritional and health implications of reducing red meat consumption must be balanced against the low certainty of evidence used to discourage red meat intake when making dietary recommendations.

## 1 Introduction

Meat-free diets are regularly encouraged as a health promotion strategy (1–3). Plant foods undoubtedly confer a health benefit, contributing soluble and insoluble fiber, micronutrients, as well as various phytonutrients and bioactive compounds, that are limited in most animal-source foods (4, 5). Similarly, animal-source foods provide certain micronutrients and bioactive compounds, including heme iron, zinc, vitamin B_12_, creatine and carnosine that are absent or have limited bioavailability in plant-source foods (4–6). For that reason, exclusion or heavy reduction of animal-source foods in favor of a primarily plant-source eating pattern may contribute to or increase the risk for certain nutrient deficiencies (7–9). Contemporary recommendations to reduce animal-source foods usually target red meat (10). The classification of animal flesh as ‘red’ is often misunderstood but is based on the concentration of myoglobin (11). According to the United States Department of Agriculture (USDA), red meat includes beef, veal, pork and lamb (12). Red meat can also encompass ‘alternate red meats’ such as venison and bison (13). Globally, beef and pork are the most consumed types of red meat (14); The concentration as well as bioavailability of some nutrients of concern are lower in non-ruminant meats (e.g., pork) when compared to ruminant meats (e.g., beef) (15–18). For example, about 72% of the iron in ruminant meat is heme iron while just 40% of the iron in non-ruminant meat is heme iron (18).

The routine consumption of red meat is often discouraged for human health, environmental sustainability, or animal welfare reasons (10). However, these are complex issues, which are often over simplified and frequently overlook the nutritional value of red meat as part of a balanced diet (19, 20). Environmental sustainability and animal welfare considerations of red meat production are beyond the scope of the present discussion, which focuses on the nutritional value and health effects of red meat.

Nutritionists are at the forefront of making diet-related recommendations, translating scientific evidence into actionable recommendations, with the goal of promoting optimal health for their clients and patients, which will vary depending upon the individual and their goals as well as the selected population. When evaluating the contribution of red meat to healthy dietary patterns, two things must be considered. First, red meat is an animal-source food. Reducing or eliminating animal-source foods, including red meat, presents challenges in meeting dietary recommendations for protein, indispensable amino acids (IAA), and vitamin B_12_ (4, 7). With respect to these nutrients, red meat as well as other animal-source foods, such as chicken, can fill the gap. Second, certain micronutrients, such as iron, are more readily available in certain types of red meat, such as beef (4). These two factors together favor the inclusion of red meat as part of a balanced dietary pattern.

The following discussion is comprised of four sections. First, we discuss meatless messaging and initiatives. Then we compare the protein content of animal versus plant-source foods. Next, we examine the role of animal-source foods, particularly red meat, in balanced diets for various population groups. Finally, we evaluate the strength of the evidence in favor of reducing or eliminating red meat due to health concerns.

## 2 Meatless messaging

In recent years, meatless initiatives have gained popularity (21). Several United States schools have initiated campaigns to remove red meat as well as poultry, pork, and seafood from the menu on one or more days of the week as a measure to promote health and environmental stewardship (22–25). There is consensus that meals containing animal-source foods carry a higher carbon footprint than plant-source meals, with red meat being the highest contributor (26–28). While these initiatives do have merit, the possibility that the exclusion of red meat could exacerbate nutrient deficiencies, such as iron or zinc deficiency, among vulnerable children must be acknowledged (29). A recent investigation found that zinc content was significantly lower in vegetarian meals compared to non-vegetarian meals (27). Although iron content was not found to be significantly different, heme iron content was not evaluated (27). The difference between heme iron versus non-heme iron, bioavailability, and iron status is discussed in the micronutrient section.

Meatless initiatives are not confined to schools. Government subsidized programs such as Women, Infants and Children (WIC) offer financial assistance for tofu and eggs, but meat products (except red meat or poultry in single ingredient baby foods) are not eligible (30). Many New York City hospitals encourage vegan meals over meals containing animal-source foods, and this approach is advocated as a best practice for all hospitals (31, 32). In addition, some advocacy groups encourage near elimination of meat (including red meat) or its taxation as a matter of public policy (33, 34). An unintended consequence is that nutritional adequacy could suffer, due to reduced intake of red meat inside of hospitals and upon discharge due to a perception that red meat is unhealthy (35). Notably, filling nutrient gaps created by the exclusion of red meat may not be as simple as supplementation because adherence and bioavailability are variable (36–38).

## 3 Protein content of animal and plant-source foods

Plant-source foods are of lower protein quality compared to animal-source foods because plant-source foods are low in one or more IAAs and the bioavailability of these essential nutrients is lower (39, 40). In addition, meat (including lean red meat) provides more protein per weight and relative to total kilocalories than plant-source foods (Table 1). While tofu is considered a high-quality vegan source of protein, the density of certain micronutrients, such as zinc and vitamin B_12_, is lower than beef (Table 1). The dark meat from turkey is a good source of iron, meaning that it provides at least 10% of the Recommended Dietary Allowance (RDA) (4). When compared to red meat, other non-red meat sources within the category of animal-source foods, such as milk and chicken breast, are lower in bioavailable iron as well as zinc and vitamin B_12_ (Table 1). Older adults and young children, who may have difficulty consuming large volumes of food (41–44), could benefit from the protein and micronutrient density of red meat, as could individuals focused on weight management.

**TABLE 1:** Protein and micronutrient values based on 100 g serving of selected animal and plant source foods^1^.

	Beef, top sirloin, steak, separable lean only, trimmed to 0” fat, select, cooked, broiled (16)	Beef, ground, 90% lean meat / 10% fat, crumbles, cooked, pan-browned (17)	Chicken, broiler or fryers, breast, skinless, boneless, meat only, cooked, grilled (116)	Milk, nonfat, fluid, with added vitamin A and vitamin D (fat free or skim) (87)	Beans, kidney, red, mature seeds, cooked, boiled, without salt (117)	Tofu, raw, firm, prepared with calcium sulfate (118)	Eggs, Grade A, Large, egg whole (84)
Energy (kcal)	177	230	151	34	127	144	143
Protein (g)	30.80	28.40	30.50	3.37	8.67	17.30	12.4
Iron (mg)	1.92	3.08	0.45	0.03	2.94	2.66	1.67
Vitamin B_12_ (mcg)	1.47	2.8	0.21	0.50	0	0	1.02
Zinc (mg)	5.7	6.84	0.9	0.42	1.07	1.57	1.24
**Protein and micronutrient values based on 100 kilocalories of selected animal and plant source foods^2^**							
Mass (g)	56	43	66	294	79	69	70
Protein (g)	17.40	12.35	20.20	9.91	6.83	12.01	8.67
Iron (mg)	1.08	1.34	0.30	0.09	2.31	1.85	1.17
Vitamin B_12_ (mcg)	0.83	1.22	0.14	1.47	0.00	0.00	0.71
Zinc (mg)	3.22	2.97	0.60	1.24	0.84	1.09	0.87

^1^Protein and micronutrient values based on 100 g serving per the USDA Food Data Central (16, 17, 41–45).

^2^Value per 100 kilocalories was calculated based upon values reported by the USDA (16, 17, 84, 87, 116–118) of the related nutrient per 100 g.

Protein intake above the current RDA has been shown to preserve lean mass during kilocalorie restriction (45). While recommended protein needs and IAA requirements can be met on a vegan diet, additional kilocalories accompany the increased amount of food needed (7). A vegetarian meal pattern can match the total protein and energy provided by an omnivore diet (7), but other factors, including micronutrient density, may make inclusion of lean red meat the more nutritionally balanced option (Table 1). Consuming sufficient high-quality protein to maintain muscle mass and promote weight loss as well as adequate micronutrients to maintain health requires nutrient dense, kilocalorie-conscious meal patterns. The inclusion of foods that are both protein and micronutrient dense, such as lean red meat, facilitates this approach.

### 3.1 Potential benefits of lean red meats for individuals experiencing obesity

In the United States, approximately 42% of adults and 20% of children are obese (46, 47). Including protein-rich animal sources like red meat in a weight management diet can be beneficial. Red meat provides satiating protein and essential nutrients, making it a suitable option for those monitoring calorie intake (48). Individuals focused on weight management must be cognizant of the fat content in red meat. For example, Hall et al. reported that *ad libitum* energy intake was significantly higher among participants consuming a high-fat animal-source ketogenic diet compared to the *ad libitum* energy intake among participants consuming a low-fat plant source diet (49). Since fat is the most energy-dense macronutrient, lean red meats may be preferred in populations focused on weight management (4).

With respect to protein density, a recent meta-analysis reported that overweight or obese participants following a dietary intervention with increased protein (18–59% of kilocalories) lost an additional 1.6 kg (95% CI: 1.2–2 kg) when compared to controls following another weight management strategy (50). Among individuals with obesity, modest weight loss (5–10%) improves health outcomes (51), and several pounds of additional weight loss is likely clinically meaningful. In addition, the inclusion of lean red meat may offer benefits irrespective of reductions in weight. Despite maintaining their baseline weight, obese participants following a Mediterranean dietary pattern that included lean beef or pork (17 ounces per week) decreased their LDL and total cholesterol significantly more than the group consuming an isocaloric amount of lean poultry (15 ounces per week) (52). This result supports the potential benefit of including lean red meats in the context of a healthy dietary pattern, such as the Mediterranean diet for weight management initiatives.

### 3.2 Potential benefits of lean red meats during pregnancy

Pregnancy is a critical period of growth and development during which malnutrition can result in long term negative health consequences (53, 54). Protein is of particular importance due to its essential role in cognitive development and linear growth59). Evidence from Elango and Ball suggests protein requirements during pregnancy may exceed current recommendations (55). Pregnant women are more likely to meet their protein needs if they consume protein from animal sources, such as red meat, poultry, and seafood and as their percentage of energy from these animal-source foods increases (56).

### 3.3 Potential benefits of lean red meats during childhood

Adequate protein intake is essential for growth and cognitive development during early childhood (57). Hovinen et al. reported that circulating amino acids were significantly lower among vegan children when compared to omnivorous children, even though protein intake relative to total energy appeared adequate (at least 10% of kilocalories) (58). This observation emphasizes the significance of protein quality in routine eating patterns. As mentioned above, most plant sources of protein do not contain the full spectrum of IAAs in adequate quantities (39, 40). Indeed, findings from Hovinen et al. underscore the importance of protein quality (58). United States children (ages 2 through 13) generally meet national dietary guidelines for protein (42). Certain segments of the population, such as adolescent girls (ages 14 through 18 years), do not meet protein recommendations (42). The recommendation for adolescent girls is to consume about 5-to-6.5-ounce equivalents of protein foods per day, whereas the average consumption among this age group approximates 4-ounce equivalents of protein per day (42). Therefore, recommendations to further reduce consumption of red meat for this population potentially increases risks for insufficient intake of essential nutrients.

Few randomized controlled trials have specifically sought to evaluate the effect of consuming protein and micronutrients derived from red meat on child health outcomes, such as cognitive performance and growth (59). Furthermore, results have been equivocal, and most of the research has been conducted in Kenya, which further limits the generalizability of the findings (59). For example, supplementation with a beef-based snack, among Kenyan children 6–14 years of age, did not result in greater height gains (compared to the nonfeeding control or isocaloric snack with milk or fat), but children supplemented with beef did experience greater gains in lean mass (60). Children receiving the beef-based snack also outperformed all other groups on measures of cognitive performance (60). Notably, the group receiving the milk-based snack fared significantly worse on measures of cognitive performance compared to all other groups, including the non-feeding control group (60). In contrast, Huelett et al. reported a significant improvement in English test scores when a snack with beef replaced an isocaloric amount of milk, but this improvement was not observed for other academic subjects, such as arithmetic (61). While additional research is needed across age groups of children and in different settings to fully elucidate the impact of red meat on cognitive development and growth in children, the aforementioned findings provide a rationale for caution in implementing eating patterns devoid of red meat in this population.

### 3.4 Potential benefits of lean red meats for older adults

Age-related declines in protein synthesis necessitate increased protein intake to manage sarcopenia, (62, 63) and strength and functionality of older adults may be improved from protein intake above the RDA (56). Mitchell et al. demonstrated that older males consuming protein above the RDA (1.6 g/kg) had greater trunk lean mass than those consuming the RDA (0.8 g/kg) for protein (64). Likewise, a recent randomized cross-over trial demonstrated higher muscle protein synthesis following a beef containing meal versus an isonitrogenous and isocaloric plant-based meal in the 6 h following the meal among males and females 65–85 years old (65). Currently, protein intakes ranging from 1.2 to 1.7 g/kg/day are recommended for older men and women (66). Notably, some advocacy groups for older adults suggest replacing meat with plant-source protein options (2). This is potentially problematic because plant-source foods cannot match the protein content of animal-source foods, including red meat, for the same kilocalories or bioavailability of key micronutrients such as iron (4, 5, 7). The combination of red meat’s protein and micronutrient density offers a means for older adults to simultaneously meet their protein needs as well as their requirements for critical micronutrients, which are discussed below.

## 4 Micronutrients of concern

Infants and young children 6–23 months, women of reproductive age, and pregnant women have increased iron requirements per calorie, while older adults have increased zinc and vitamin B_12_ requirements per calorie (67). Estimates indicate that more than half of children aged under 5 years and two-thirds of women aged 15–49 years have at least one micronutrient deficiency (68), while global data are too scarce to provide estimates for men or older adults. Children consuming an exclusively plant-source diet are at greater risk for micronutrient deficiencies (58). Contrary to the position of the United States based Academy of Nutrition and Dietetics (69), numerous pediatricians and nutritional or pediatric organizations as well as a recent systematic review, discourage vegan diets or both vegan and vegetarian diets for young children and pregnant women because the risk of micronutrient deficiencies, particularly iron, zinc, and vitamin B_12_ is elevated (70–77). Specific concerns for iron and zinc are considered in the following paragraphs. Vitamin A deficiency is also pervasive in young children globally (78). While certain animal-source foods such as chicken skin and liver are good sources, Vitamin A is not found in muscle meat in sufficient quantities to attenuate deficiency and therefore, is not considered in this perspective (4, 5).

### 4.1 Iron

Iron deficiency is common worldwide and is a major cause of anemia (78). Globally, almost 30% of women of reproductive age and almost 40% of children under the age of five were anemic in 2019 (79). Insufficient iron consumption during pregnancy is a concern specifically related to low red meat intake (6, 79). Over 80% of Irish pregnant women are estimated to be iron deficient by their third trimester (80). In the United States, the prevalence of iron deficiency among women aged 15–49 years was over 20% for the period 2015–2016 (68) and has been increasing over the past 15 years, in part due to decreased beef intake and replacement with chicken (81).

Inadequate iron intake is also high among adolescent boys in the United States (82). Heme iron, exclusively found in foods of animal origin, is two to three times more bioavailable than the non-heme iron found in plants (4). Due to these differences, vegetarians need to consume nearly twice the dietary iron to meet their needs when compared to an individual consuming an omnivorous diet (5). Pawlak et al. reported that across studies, the prevalence of iron deficiency was higher among vegetarians, a finding that highlights the aforementioned differences in iron source (83). Eggs and dairy products are the two categories of animal-source foods that may be included in a vegetarian diet (4). Depending upon an individual’s lifecycle stage (4) eggs may be a good source of iron, but it’s mostly non-heme iron and the bioavailability is limited (84–86). Dairy products are a poor source of iron for all segments of the population (4, 87).

### 4.2 Zinc

In the United States, approximately 3.8% of children under the age of 10 years and 8% of individuals 10 years of age and older are zinc deficient, respectively (88). Furthermore, in the United States zinc deficiency is estimated to be higher (14%) among women 15–49 years of age (68), and may be even higher among men, given their higher dietary zinc inadequacy (82). Zinc deficiency is less prevalent among those who consume animal-source foods (89). Vegetarians and vegans require additional dietary zinc (4) and are also at increased risk for zinc deficiency (90). This is because the phytates found in plants limit the intestinal absorption of zinc (4, 5). Zinc deficiency may be particularly problematic for young children, who need adequate zinc to maintain normal growth and development (91). Second to oysters, ruminant red meat is one of the best animal sources of zinc (88). in total, red meat can be an important source of iron, zinc, and vitamin B_12_ across critical life stages (67).

## 5 Strength of the evidence in support of anti-meat messaging

Anti-meat messaging, specifically recommendations to reduce or eliminate red meat, are based on concerns for the environment, animal welfare, and health. Concerns for environmental sustainability and animal welfare are complex and nuanced topics (19, 20) beyond the scope of this perspective, which focuses on potential health consequences of excluding red meat from dietary patterns. Depending upon the cut and type of red meat, approximately 30–50% of the total fat in red meat is saturated (92, 93), and there remains ongoing debate concerning the role that saturated fats play in cardiovascular disease (94–97). However, the United States Dietary Guidelines allow saturated fats to comprise 7–10% of total kilocalories, and red meat can be included in healthy eating patterns while staying within those guidelines (42).

Health-based recommendations to reduce or eliminate meat are largely based on research documenting a relationship between all red meats or processed meats with morbidity and mortality (98–103). However, in-between study heterogeneity is large and the certainty of the evidence implicating red meat in the development of chronic disease is low to very low, whereas, the absolute risks related to elevated consumption are small (104–106). When included as part of a healthy dietary pattern, unprocessed red meat has been associated with lower risk of micronutrient deficiencies (107, 108), improvement of cardiometabolic risk factors (52), and attenuation of sarcopenia in older adults (63). In addition, You et al. reported that red meat consumption correlated with reductions in child mortality and longer life expectancies in all areas studied (covering 90% of the globe), except for Southeast Asia (109). Notably, the EAT-Lancet diet, which recommends large reductions in red meat, does not meet requirements for certain micronutrients including iron, zinc, and vitamin B_12_ (110). Policy makers, health professionals, and advocates must consider the quality of evidence against red meat as well as the potential unintended consequences that could result from the reduction or exclusion of red meat when developing (or ‘establishing’) policies and dietary recommendations.

## 6 Summary and recommendations

Globally, the average person consumes an estimated 51 g (less than 2 ounces) of unprocessed red meat per day (111). In the United States, the estimate is 34 g (just over 1 ounce) (111). The World Cancer Research Fund and the American Institute for Cancer Research guidelines allow up to 2.5 ounces (70.9 g) of unprocessed red meat per day (112), yet consumers are routinely confronted with messages to not only reduce red meat intake below current consumption levels but to eliminate this whole food from their diets (1–3, 22–24, 31–33, 113–115). The present perspective proposes that this messaging could cause more harm than good to public health if implemented in public policy or dietary guidelines. Individuals at risk for undernutrition and obesity may benefit from increased intake of key nutrients by eating animal-source foods, including red meat. Red meat provides highly bioavailable forms of many essential nutrients of concern, such as iron, zinc, and protein. In addition, red meat is a source of these nutrients at a lower energy density and in a smaller serving size than many plant-source options. Messaging to children and other at-risk populations indicating red meat is unhealthy discourages a protein- and micronutrient-dense whole food option. The evidence that red meat is causally associated with chronic disease is mixed. Therefore, additional research is warranted to determine whether noted associations between red meat and chronic disease represent true relationships and in what amounts, or if other diet and lifestyle factors account for the variance in health outcomes. At the macro, as well as individual levels, policy makers and dietitians must weigh the nutritional profile and benefits of red meat consumption against the low certainty of evidence used to discourage red meat intake when making public health and nutrition recommendations. This approach represents a contemporary challenge for nutrition professionals endeavoring to optimize the health of their clients and patients.

## Data Availability

The original contributions presented in this study are included in this article/supplementary material, further inquiries can be directed to the corresponding author.
